# Hospital Expenditure.—The Commissariat

**Published:** 1895-12-21

**Authors:** 


### The Institutional Workshop.

### HOSPITAL EXPENDITURE.—THE COMMISSARIAT.

### X.—THE SERVANTS' TABLE.

There is perhaps less variation in the cost of the servants' board in institutions than in that of any other class of inmates. The universal requirements are that it should be plain, wholesome, and sufficient, and on these lines any number of persons may be boarded at an average cost, remaining undisturbed from year to year, and in one institution after another, except by alteration in prices.

It is the custom, even in hospitals where the accounts are analysed, and the difference in cost of different members of the household is recognised, to class nurses and servants together in the estimate of expenditure. The sisters, nurses, clerks, and male and female servants are roughly averaged together, although admittedly the servants' table is more plainly provided. The distinctions are deemed too trifling to be worth reckoning. We have already pointed out in the case of the doctors' table that trifling relaxations from the rule of rigid economy may easily double the cost of board; and as an example of the difference in cost produced by really minute distinctions of diet we cannot do better than present the diet table of the Marylebone Infirmary—carefully arranged on a scale of liberality—stating the weekly amounts allowed for nurses and servants respectively, and reckoning the difference in cost between the one class and the other.

| Nurses. | Servants. |
|---|---|
| 7 lb. bread ... ... ... ... | Do. |
| 7 lb. meat (uncooked, including bone) | Do. |
| 7 lb. potatoes ... ... ... ... ... | Do. |
| 1 lb. flour... ... ... ... ... ... | Do. |
| 1 lb. rice, or ½ lb. currants, tapioca, sago | Do. |
| ¾ lb. butter ... ... ... ... ... | Do. |
| ¾ lb. bacon ... ... ... ... ... | Do. |
| ½ lb. cheese ... ... ... ... ... | Do. |
| 1 lb. loaf sugar ... ... ... ... ... | ½ lb. |
| ½ lb. moist sugar ... ... ... ... | Do. |
| 7 pints milk ... ... ... ... ... | 3½ pints. |
| 7 eggs ... ... ... ... ... ... | 4 eggs. |
| 4 oz. tea ... ... ... ... ... ... | Do. |
| 8 oz. coffee ... ... ... ... ... | 4 oz. coffee. |
| 3 oz. jam ... ... ... ... ... ... | Do. |
| Fish once a week ... ... ... ... | Do. |
| Vegetables daily ... ... ... ... | 1½ lb. veg. |
| 7 pints ale, milk, soda water, or non-alcoholic ale ... ... ... | Do. |
| Pepper, mustard, vinegar, and salt as required ... ... ... ... | Do. |
| Pickles, sauces, fruit for cooking as required ... ... ... ... | Fruit for cooking once a week. |

The scale probably represents merely the maximum consumption authorised, and as the meals are prepared together the cost per head is considerably less than appears to be the case if each item is priced separately, and all are added together. The difference in the servants' board can hardly be less than 2s. a week, reckoning the extra fruit, sauces, &c., of the last items at no more than 2d. a day. In practice the cost of the nurses' food at Marylebone Infirmary works out at about 8s. a week a-head, and this would place the servants' board at 6s. a-head. This may be considered a safe average for the servants' table in institutions where the diet is on a liberal scale—where meat, or substitute for meat, is provided three times daily, and malt liquor is included. The small additions which go to make the nurses' diet rather more varied and attractive, taken together, cannot be estimated at less than from 3d. to 6d. a day per head in most institutions.

Under the allowance system a larger quantity of meat and drink is commonly assigned to male servants. For instance, in the Sussex County Hospital, the diet table of which has been already quoted, the porters receive 3 oz. of extra meat and an extra quart of beer daily, the cost of which is at least 2s. 4d. a week.

The servants' allowance at Westminster Hospital is daily: Bread, 1 lb.; meat, 1 lb.; milk, ½ pint; ale, 1 pint women, 2 pints men. Weekly: 4 oz. tea; ¾ lb. sugar; ½ lb. butter; 1 lb. cheese. This cannot be said to err on the side of profusion, but it is probably like the majority of diet sheets printed, more honoured in the breach than in the observance. It is to be noted that the men reckon at least 10½d. a week more than the women with their extra pint of beer. It would be safe to reckon invariably 1s. weekly a-head extra for male servants.

The allowance system is now generally discredited, and meals are usually served in common with practically no restriction as to quantity. It was no doubt often a protection under careless administration, but under the rule of a good housekeeper its abolition would be almost certainly a source of saving and of satisfaction to all concerned.

Partial board has been reckoned in these calculations as half. In many cases no doubt two-thirds would be nearer the mark, it being matter of observation that those who are thus boarded accustom themselves to make the meals consumed on the premises their principal ones. For this reason, if food is supplied at all to casual workers living outside, the allowance system is in this one case greatly to be preferred. The tacit understanding by which all that is left at a meal is divided among the scrubbers (who are not otherwise provided for) is a direct incentive to waste, and should not be tolerated in any institution household, however firmly the custom may be rooted in tradition. The knowledge that all waste in providing will directly benefit a struggling class of workers is a comfortable doctrine likely to affect very considerably the morality of the kitchen.

Looking at the question purely from a monetary point of view, it is, of course, far cheaper to pay these women by the hour and provide no board, this kind of unskilled female labour being a drug in the market, and the class which furnishes it being expensive to board. But if it be conceived that the hospital is bound to take a far higher standpoint than that of mere £ s d, it may be deemed that in providing a good meal for its underfed assistants, many of them mothers of families, it is aiding to set up in the neighbourhood a standard of just remuneration and of liberality for all around.

